# Fast-Track Liver Transplantation: Six-year Prospective Cohort Study with an Enhanced Recovery After Surgery (ERAS) Protocol

**DOI:** 10.1007/s00268-021-05963-2

**Published:** 2021-02-23

**Authors:** Gonzalo P. Rodríguez-Laiz, Paola Melgar-Requena, Cándido F. Alcázar-López, Mariano Franco-Campello, Celia Villodre-Tudela, Sonia Pascual-Bartolomé, Pablo Bellot-García, María Rodríguez-Soler, Cayetano F. Miralles-Maciá, Patricio Más-Serrano, José A. Navarro-Martínez, Francisco J. Martínez-Adsuar, Luis Gómez-Salinas, Francisco A. Jaime-Sánchez, Miguel Perdiguero-Gil, María Díaz-Cuevas, José M. Palazón-Azorín, José Such-Ronda, Félix Lluís-Casajuana, José M. Ramia-Ángel

**Affiliations:** 1 Hepatobiliary Surgery and Liver Transplantation, Hospital General Universitario de Alicante, Alicante, Spain; 2 Hepatology and Liver Unit, Hospital General Universitario de Alicante, Alicante, Spain; 3 Pharmacy and Pharmacokinetics, Hospital General Universitario de Alicante, Alicante, Spain; 4 Anesthesiology and Surgical Critical Care, Hospital General Universitario de Alicante, Alicante, Spain; 5 Critical Care Medicine, Hospital General Universitario de Alicante, Alicante, Spain; 6 Nephrology and Renal Transplantation, Hospital General Universitario de Alicante, Alicante, Spain; 7 Anesthesiology and Surgical Critical Care, Hospital Marina Baja, Villajoyosa, Alicante, Spain; 8 Digestive Disease Institute, Cleveland Clinic Abu Dhabi, Abu Dhabi, United Arab Emirates; 9 ISABIAL (Alicante Institute for Health and Biomedical Research, Alicante, Spain

## Abstract

**Introduction:**

Enhanced recovery after surgery (ERAS) has been shown to facilitate discharge, decrease length of stay, improve outcomes and reduce costs. We used this concept to design a comprehensive fast-track pathway (OR-to-discharge) before starting our liver transplant activity and then applied this protocol prospectively to every patient undergoing liver transplantation at our institution, monitoring the results periodically. We now report our first six years results.

**Patients and methods:**

Prospective cohort study of all the liver transplants performed at our institution for the first six years. Balanced general anesthesia, fluid restriction, thromboelastometry, inferior vena cava preservation and temporary portocaval shunt were strategies common to all cases. Standard immunosuppression administered included steroids, tacrolimus (delayed in the setting of renal impairment, with basiliximab induction added) and mycophenolate mofetil. Tacrolimus dosing was adjusted using a Bayesian estimation methodology. Oral intake and ambulation were started early.

**Results:**

A total of 240 transplants were performed in 236 patients (191♂/45♀) over 74 months, mean age 56.3±9.6 years, raw MELD score 15.5±7.7. Predominant etiologies were alcohol (*n *= 136) and HCV (*n *= 82), with hepatocellular carcinoma present in 129 (54.7%). Nine patients received combined liver and kidney transplants. The mean operating time was 315±64 min with cold ischemia times of 279±88 min. Thirty-one patients (13.1%) were transfused in the OR (2.4±1.2 units of PRBC). Extubation was immediate (< 30 min) in all but four patients. Median ICU length of stay was 12.7 hours, and median post-transplant hospital stay was 4 days (2-76) with 30 patients (13.8%) going home by day 2, 87 (39.9%) by day 3, and 133 (61%) by day 4, defining our fast-track group. Thirty-day-readmission rate (34.9%) was significantly lower (28.6% vs. 44.7% *p*=0.015) in the fast-track group. Patient survival was 86.8% at 1 year and 78.6% at five years.

**Conclusion:**

Fast-Tracking of Liver Transplant patients is feasible and can be applied as the standard of care

## Introduction

Enhanced recovery after surgery (ERAS) is affected through multiple steps, many of which have been independently studied. However, there are few studies that have evaluated the effects of protocols that incorporate previously established and validated interventions into a single compendium applied to liver transplant recipients. Furthermore, these commonly referred to as “fast track” protocols are not routinely used at new centers since a learning curve is thought to be a prerequisite for the successful execution of the involved steps.

The present study has two main objectives: 1) to evaluate the effects of a protocol that incorporates previously established and validated fast-track interventions into a single application and 2) to use this protocol from the beginning of a new program to determine whether such a multistep fast-track protocol can be successfully applied by a group without any previous learning period.

In the present study, the term “fast track” entails a comprehensive approach to the entire admission event, from the moment the patient sets foot in the hospital until the time of discharge. It integrates several perioperative steps (maneuvers and techniques) most of which are already well-established and widely utilized, aimed at minimizing hospital stay without compromising patient’s safety [1, 2]. For the purpose of our protocol, we considered optimization of anesthesia, refinement in surgical technique, minimization of blood loss, precise intraoperative coagulation management, early extubation, aggressive postoperative rehabilitation with early oral nutrition and ambulation, a personalized immunosuppression, and adequate pain control.

## Patients and methods

This is a prospective cohort study, designed back in 2011, before the start of a new liver transplant program. We created a multidisciplinary team to devise a fast-track pathway that could be applied to our entire liver transplant population. The team included surgeons, anesthesiologists, hepatologists, critical care physicians, pharmacists, radiologists, nephrologists and nursing staff. The purpose was to apply all the available and proven knowledge in early extubation and short ICU stay, and expand it to include an aggressive recovery protocol to allow for a quicker passage through the surgical ward and onto discharge. The present study reports our results after our first six years (2012–2018) utilizing this fast-track pathway on all recipients of a cadaveric liver (and combined liver and kidney) transplant at our institution with a minimum follow-up of one year. There was no funding involved in the present study.

All patients were included in the study and were listed following the implementation of a sickest-first prioritization-based allocation system using the Model for End-Stage Liver Disease (MELD) score, with hepatocellular carcinoma (HCC) weighing. Donor information included age, sex, cold ischemia time—defined as the time from cross-clamping in the donor to the removal of the organ from the cold preservation solution, warm ischemia time—defined as the time elapsed from the end of the cold ischemia until the reperfusion of the graft—, and donor risk index [3].

General anesthesia was induced with propofol (1.5–2.0 mg/kg), rocuronium (1 mg/kg) and fentanyl (2–3 μg/kg), and maintained with sevoflurane, remifentanyl (0.2 mcg/kg-min) and rocuronium (5 mcg/kg-h). Two indwelling catheters were placed in left radial and right femoral arteries. Two high-flow catheters were routinely placed in the left basilic and right jugular veins, and a pulmonary artery catheter (PAC) was placed when deemed necessary. Fluid restriction was implemented throughout the surgical procedure. Plasmalyte® was given at a rate of 2–4 ml/kg-h to cover for unaccounted fluid losses and albumin for blood losses at a ratio of 1:1. Packed red blood cells (PRBC) transfusions were given for hemoglobin <7 g/dl, and/or venous oxygen saturation <70% (central or mixed). Routine preoperative hemoextraction (whole blood) was performed when preoperative hemoglobin ≥9 g/dl. The maximum amount drawn was three units, and extraction was stopped when venous oxygen saturation <70%. The blood was stored at room temperature in the operating room and reinfused during the biliary reconstruction. Cell saver was used in patients who did not have a diagnosis of HCC or any intraoperative evidence of contamination or infection.

The hemodynamic management was adjusted using the PAC or the Pulse index Continuous Cardiac Output (PiCCO^®^, Pulsion) based on volumetric preload parameters such as global end-diastolic volume (GEDV). We aimed at maintaining a mean arterial pressure of 65 mmHg, for which norepinephrine was used as needed. We added Terlipressin (1mg) boluses in patients with advanced cirrhosis and ascites to facilitate the weaning from norepinephrine.

The standard surgical technique included preservation of the inferior vena cava (VCP) and a temporary portocaval shunt (TPCS). The grafts were washed prior to reperfusion with 500 ml of a balanced intravenous electrolytic solution (PlasmaLyte or Ionolyte) through the portal vein at room temperature. Blood flows were recorded intraoperatively by transit time flow measurement (Medistim ASA, Oslo, Norway) on the native portal vein, the TPCS (after its completion and prior to its closure) and in the portal vein and hepatic artery after reperfusion and just prior to abdominal closure. In order to minimize biliary ischemia in older donors and donors after cardiac death (DCD), we decided to perform either arterial or simultaneous arterial and portal reperfusion in cases with donors 70 years and older and all DCDs. All biliary reconstructions were performed duct-to-duct without T-tube, except for patients with primary sclerosing cholangitis who underwent Roux-en-Y hepaticojejunostomy.

Coagulation disorders present at the start of the procedure were not corrected; instead, we used thromboelastometry and/or thromboelastography (TEG) throughout the operating time as a guide to correct coagulopathy and help optimize blood products usage. Analgesia was managed with remifentanyl, and a bolus of morphine (0.1 mg/kg) given right after reperfusion. Sugammadex was given at the end of the procedure to reverse the neuromuscular blockade. Early extubation was defined according to Mandell [4] as removal of the endotracheal tube immediately following surgery (up to 6 hours). We followed standard criteria for extubation: reversal of neuromuscular blockade judged by peripheral nerve stimulator and clinical assessment, patients following verbal commands, positive gag reflex, tidal volumes >6 ml/kg, respiratory rate <20, oxygen saturation >95% while breathing spontaneously (FiO2 ≤50%), normocarbia assessed by end-tidal CO_2_, and core body temperature between 36.5 and 37.5 °C.

Following extubation, patients were transferred to either the medical or the surgical ICU with continuous monitoring. Logistics included the availability of a dedicated physician and nurse for the immediate postoperative period (6–8 hours) and 1:1 or 1:2 nursing to patient ratio thereafter until ICU discharge. Oral intake was started liberally 4 hours after arrival to the ICU, and abdominal Doppler ultrasonography was performed within the first 12 hours. Before the transfer to the surgical ward, arterial lines and pulmonary artery catheters (when used) were removed. The surgical ward, a 27–30 bed nursing unit dedicated to patients before and after general surgery, has a 1:10 nurse-to-patient ratio. Patients were monitored initially by continuous pulse oximetry and noninvasive blood pressure measurements. Urine output was measured 3 times daily, and body weight and abdominal perimeter were obtained daily. Patients started ambulating early, most commonly on the very transplant day.

### Standard immunosuppression was achieved with a regime of

a Steroids: methylprednisolone 500 mg IV given immediately after full revascularization of the graft (once the portal and arterial inflows were reestablished) followed by a 4-day IV and 8-day oral stepwise dose reduction until day 12th, with prednisone 10 mg PO daily until post-transplant day 90, and prednisone 5 mg PO daily until day #180, when they are discontinued.
b Tacrolimus (Advagraf® - its prolonged-release form) 0.1–0.15 mg/kg PO once-daily.
c Mycophenolate mofetil (1 g PO /12 h).
d Basiliximab (Simulect®) was used for induction in patients with renal dysfunction, followed by a delayed start of Advagraf.

An intensive pharmacokinetic monitoring program was implemented right after the first dose of Advagraf, drawing blood samples after the first dose to measure T_2_ (2 hours post-dose), T_3_, T_5,_ T_12_ and trough level (T_0_). Blood samples were immediately assayed using an auto-analyzer (QMS® technology, Indiko® platform—ThermoFisher®). The dose of tacrolimus to achieve a given target steady plasma level was calculated using a Bayesian estimation methodology based on pharmacokinetic individual parameters. Target levels were chosen for each patient according to liver and kidney function and the etiology of their liver disease. We also obtained daily trough levels until discharge and during regular visits to the outpatient clinic. In addition, a mini curve (T_0_, T_2_ and T_3_) was obtained on days 15th and 30th after liver transplantation, at 2, 3 and 6 months, and on demand whenever deemed necessary to calculate the area under the curve and individualize the dosing of both tacrolimus and MMF.

Hospital discharge was decided upon a tendency toward normalization of liver and kidney function, independent ambulation, and self-adherence to treatment. Outpatient follow-up was initially twice a week and attended simultaneously by hepatologists, surgeons and pharmacists. For a better visualization of our fast-track protocol, a summary checklist is outlined in Table 1.

**Table 1:** Itemized checklist of our fast-track protocol

Pre-Transplant (outpatient evaluation)	
1	A suitable environment is available for immediate discharge after transplant
2	Adequate support from family (and/or others)
3	Patient and relatives understand the Peri- and Post-transplant process
4	Direct communication phone list available

Transplant hospital admission	
5	Cleansing enema
6	Antiseptic (povidone–iodine or chlorhexidine) soap shower
*Intraoperative*	
7	Standard procedure with IVC preservation and Temporary Portocaval Shunt
8	Blood sparing techniques: hemoextraction, TEG, cell saver. Aim at avoidance of blood products
9	Adequate pain control: morphine bolus post-reperfusion
10	Minimal ionotropic support by the end of the surgical procedure
11	Patient wakes up and follows commands
12	Standard extubation criteria are met
	Presence of gag reflex
	Tidal Volume > 6 ml/kg
	Respiratory rate < 20/min
	SPO2 > 95% with FiO2 < 50
*Postoperative (SICU or MICU)*	
13	Improvement of blood work (working graft)
14	Finish/continue fluid replenishment. Withdrawal of ionotropic support
15	Abdominal Doppler US within the first 12 hours without anomalies
16	Incentive spirometry
17	Initiate regular diet
18	Removal of large-bore IV lines and invasive monitoring
19	Removal of urinary catheter
*Postoperative (surgical ward)*	
20	Immediate ambulation and use of chair (avoidance of bed during the daytime)
21	Start and/or complete a full pharmacokinetic immunosuppression (IS) profile (T2, T3, T5, T12, T0)
22	Tendency toward normalization of blood work (liver biochemistry and renal function)
23	Can complete shower (self-hygiene in general) and all other basic activities of daily life (BADL)
24	Patient and family education on daily activity and use of immunosuppression
25	Final education on diet, hygiene, medications, exercise and contact with the transplant team

Post-Transplant (outpatient follow-up)	
26	Initial outpatient visits with blood work twice weekly
27	Reduced pharmacokinetic IS profile (T2, T3, T0)
28	US or CT scan on demand

Statistical analysis. Categorical variables are reported as frequency or percentages. Descriptive statistics for normally and non-normally distributed continuous data are reported as mean ± standard deviation (SD) or median with range (lower quartile and upper quartile), respectively. Categorical variables were compared with the Chi-square test or Fisher exact test, when appropriate. Differences between groups for normally and non-normally distributed quantitative data were analyzed using the independent samples t test or the Mann–Whitney U test, respectively. The Kaplan–Meier life table was used to analyze survival and timeframe differences, and the Log-rank test was used to compare survival curves. Variables with p value lower than 0.05 are considered to be significant. All calculations were performed using SPSS Statistics (version 26, IBM Corporation). The post-transplant length-of-stay (PTLOS) analysis was performed on the patients who were discharged after transplantation.

## Results

Two hundred and forty consecutive orthotopic liver transplants were performed on two hundred and thirty-six patients (191♂, 45♀), mean age 56.3±9.6 years (range 19–70), with raw MELD score at time of transplant of 15.5±7.7 (MELD-Na 16.8 ±8.1) over a period of 74 months (Table 2). All livers were obtained from cadaveric donors, of which 16 were Maastricht category III donors after circulatory death. Mean donor age was 60.4±16.8 years (range 11–90; *P*_25-75_: 49–74). Donor risk index was 1.84±0.42 (range 0.82–3.3; *P*_25-75_: 1–2). The main etiology was alcohol (*n*=136) followed by HCV (*n*=82), HBV (*n*=14), non-alcoholic steatohepatitis (*n*=8), and primary biliary cirrhosis (*n*=7). There were 129 patients (54.7%) with hepatocellular carcinoma and 11 cases of fulminant hepatic failure. Four patients were retransplanted and nine underwent combined liver and kidney transplantation. The minimum follow-up time was one year, and no patient was lost to follow-up.

**Table 2:** Donor and recipient characteristics and demographic data

Donors	Mean±SD or N (%)
Age (years)	60.4 ± 16.8
Donor risk index	1.84 ± 0.42
Donors after circulatory death (Maastricht III) (n)	16 (6.7%)
Recipients	
Age (years)	56.3 ± 9.6
Gender (female/male)	45 (19.1%) / 191 (80.9%)
MELD (laboratory)	15.5 ± 7.7
MELD-Na	16.8 ± 8.1
Etiology	
Alcohol (all)	136 (57.6%)
Alcohol + HCV or HBV	43 (18.2%)
HCV	82 (34.8%)
HBV	14 (5.9%)
NASH	8 (3.4%)
PBC	7 (3%)
Other	32 (13.6%)
Patients with HCC	129 (54.7%)
Urgent status (Acute Liver Failure)	11 (4.7%)
Presence of ascites (n)	107 (45.3%)
Volume of ascites (ml)	3547 ± 3707
Combined liver/kidney transplant (n)	9 (3.8%)

The standard surgical technique was not applied in just two cases: one with TPCS and no VCP and another one with VCP and no TPCS. Cold ischemia time was 279±88 min (range 130–628) and warm ischemia time 42.3±7.1 min (range 27–67). Mean operating time was 315±64 min (range 167–546). Only thirty-one patients (13.1%) received packed red blood cells (PRBC) from the blood bank in the operating room, at an average of 2.4±1.2 units per patient. The average blood recovered using cell saver in the 107 patients without HCC was 571±427 ml (range 30–2000; *P*_25-75_: 232–745). We initially left abdominal drains but decided to stop using them after the first 19 cases.

All patients underwent immediate extubation in the OR but four (one with primary graft non-function, one with morbid obesity not meeting extubation criteria and two with severe encephalopathy due to their fulminant liver failure). The elapsed time between the conclusion of the procedure and patient’s extubation did not exceed 30 minutes in the entire group. The median length of stay in the ICU was 12.7 hours (range 3.7–799; *P*_25-75_: 9.4–28.3).

**Fig. 1:**
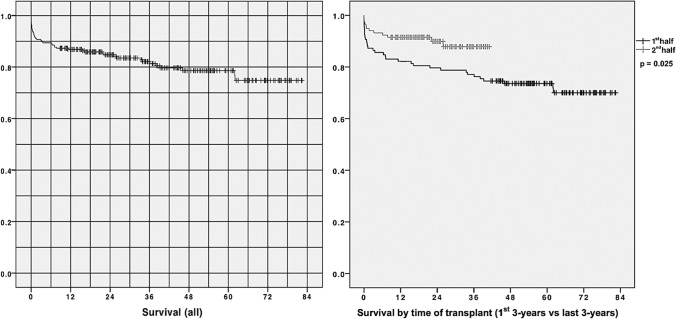
Patient survival (entire series on the left and first vs second 3-year period on the right). 139x69mm (300 x 300 DPI)

**Fig. 2:**
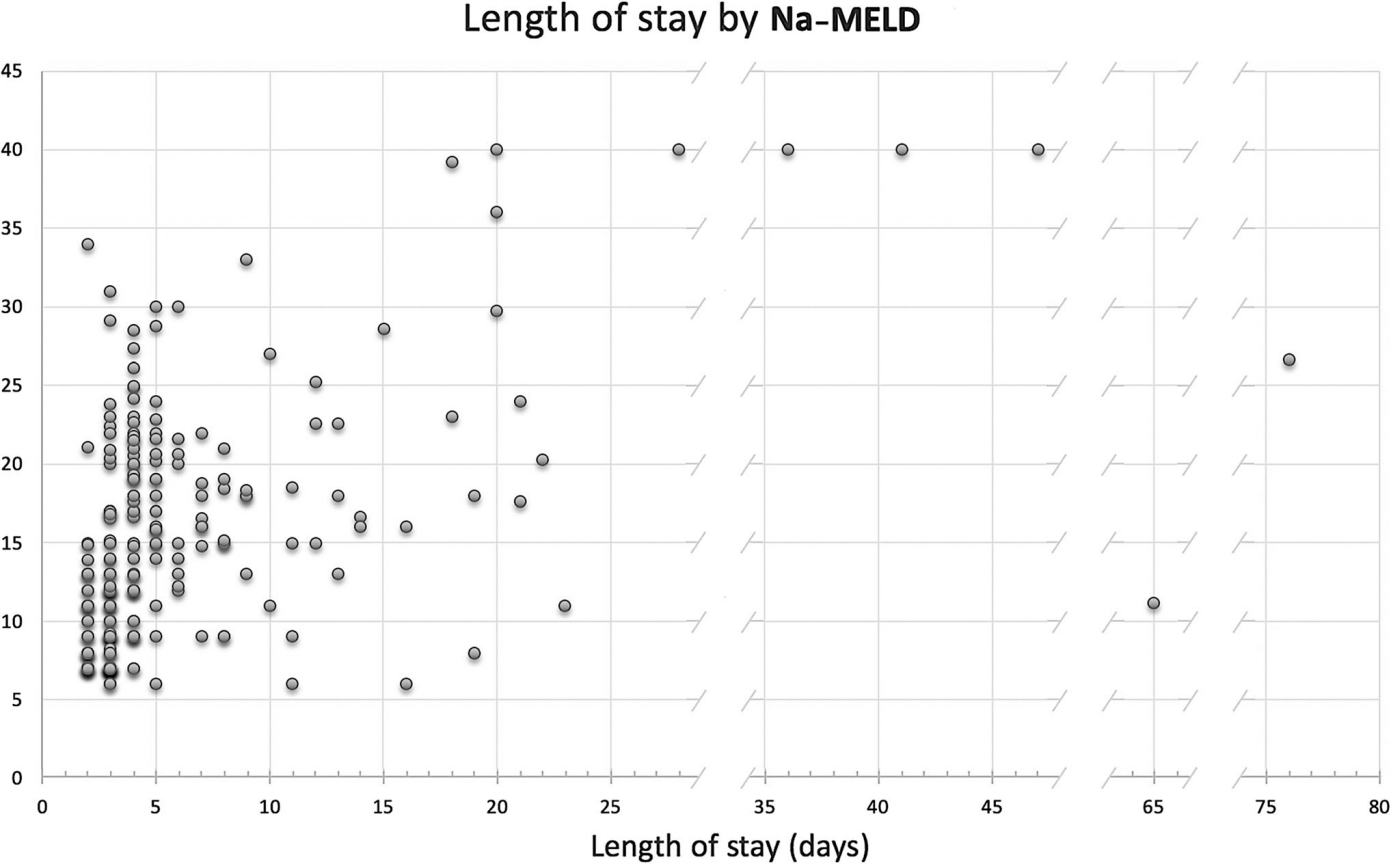
Post-Transplant LOS in days according to Na-MELD (all discharged patients). 183x117mm (300 x 300 DPI)

Our first liver transplant patient was discharged on the fourth day, and the median PTLOS (4 days) has remained unchanged ever since (range 2–76; *P*_25-75_: 3–7). We have used this median to set the boundary for our early discharge group, which includes those patients who successfully completed the fast track. Eighteen of the 236 patients died during the transplant admission (never discharged) and thus were excluded from the PTLOS analysis. The overall survival for the entire group (86.8% at 1 year and 78.6% at 5 years) is shown in the Kaplan–Meier graphs (Fig 1), and it is significantly better for the second half (last vs. first 3-year period, *p*=0.025) of our activity. Of the 218 patients discharged home after transplant, 133 (61%) were early discharges (30 on the 2nd day, 57 on the 3rd and 46 on the 4th) and they constitute the fast-track group. MELD score did not seem to predict PTLOS except for values >35, as shown in Fig 2. Regarding survival in these patients, even though there was a slight trend toward better survival at 1-year (95.5 vs. 90.5%) and 5-year (85.3 vs. 81.6%) in the fast-track group, these differences did not reach statistical significance (*p* = 0.44).

Seventy-six patients (34.9% of all the discharges) were readmitted within 30 days of their discharge. The proportion of 30-day readmissions amongst the fast-track group was significantly lower (28.6 vs. 44.7%, *p*=0.015) than that of the regular discharge group, and the rate of readmission was higher in those patients showing evidence of bacterial DNA translocation [5].

**Fig. 3:**
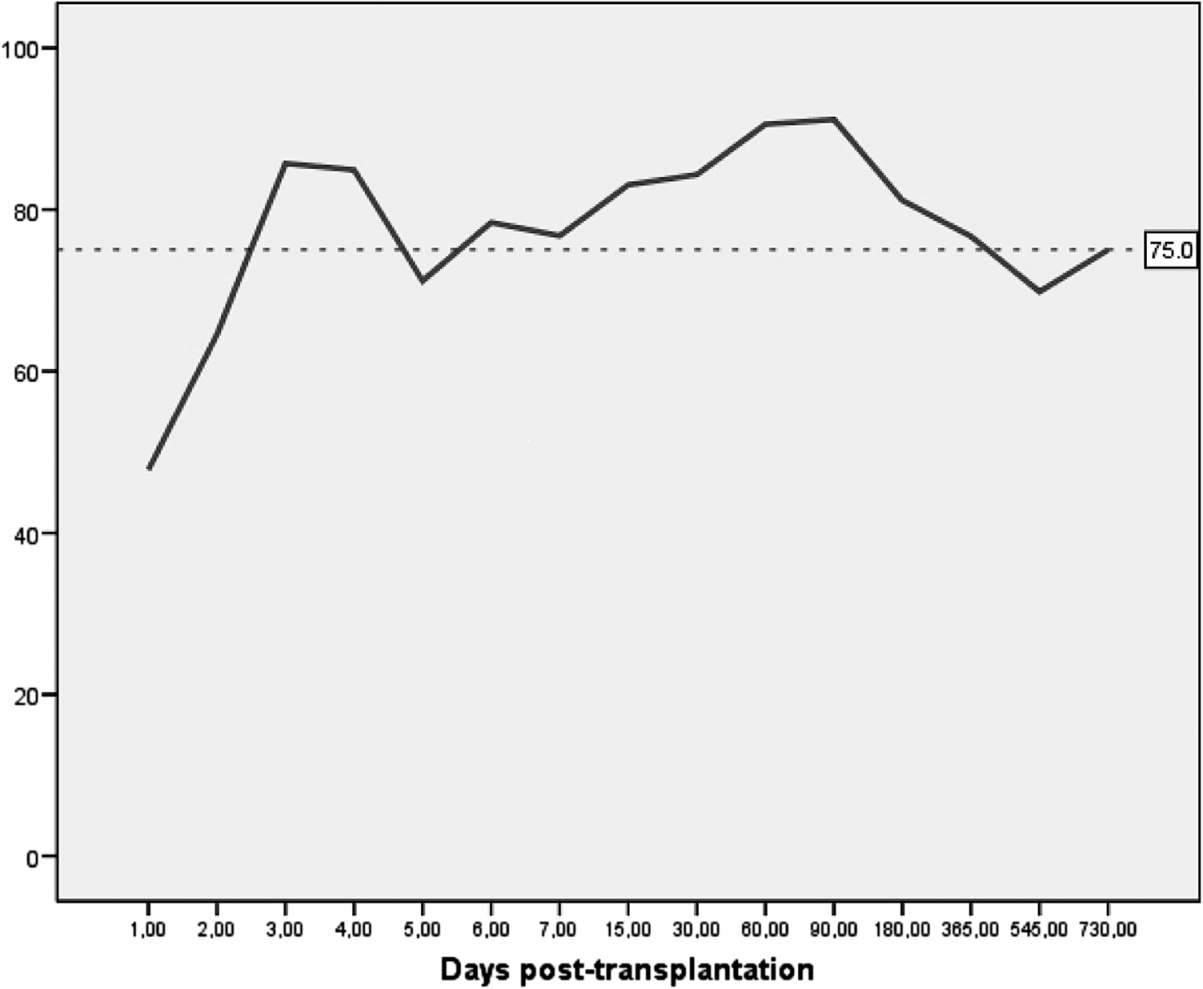
Percentage of patients with plasma levels of tacrolimus within therapeutic range after liver transplantation

Plasma concentrations of tacrolimus achieved a stable level between days 3rd and 7th following liver transplantation. Median values were 9.0 ng/ml (*P*_25-75_: 6.1–18.0) on day 3rd, 8.4 ng/ml (*P*_25-75_: 6.5–14.9) on day 7th, and 9.5 ng/ml (*P*_25-75_: 7.3–13.0) on day 15th, with target concentrations remaining stable thereafter. Although a slow decline over time was observed (median value of 9.2 ng/ml [*P*_25-75_: 6.8–12.8]) during the 1st month, versus 7.1 ng/ml [*P*_25-75_: 6.1–8.1] during the 6th month), the majority of patient-maintained levels within therapeutic range (Fig 3).

## Discussion

The present study is a thorough analysis of all the prospectively collected data since the start of our liver transplant program. We rationalized and designed our protocol based on the available knowledge. Our fast-track pathway was designed aiming at enhancing the recovery of our patients throughout their entire transplant hospital stay. The main outcome measures were length of hospital stay and survival. We monitored our results and compared them with those of similar centers across Spain [6] and elsewhere.

The protocol applied in our study concatenates multiple well-established perioperative fast-track steps, paving a speedy pathway through which most of our patients can move swiftly until discharge. There is not a single concept in the present study that has not been applied in liver transplantation before; the novelty consists in linking all those concepts together to create a comprehensive clinical pathway and showing its value as a proof-of-concept.

Previous authors have consistently demonstrated the safety and benefits of early extubation after liver transplantation [7, 8] and its positive impact on survival [9] and costs savings [10], while others have shown a reduced need for mechanical ventilation but not for ICU stay^11^. Further review of the available literature [4, 12] confirms that early extubation after liver transplantation is feasible, safe, and cost-effective, and it has been increasingly accepted as a better option over conventional postoperative ventilation due to its physiological (graft blood flow improvement, lesser complications from mechanical ventilation, improvement of patient comfort, etc) and economical (better utilization of resources, cost containment, etc) advantages. Fast tracking has also been shown to allow an altogether bypassing of the ICU after liver transplantation, providing a 1:1 nursing to patient ratio and close monitoring are in place for the initial 24 hours [13], although some concerns have been raised regarding ICU avoidance [14]. As stated previously, our protocol called for mandatory ICU admission.

Mandell [4] showed that the number of patients undergoing early extubation at different institutions can vary widely (5–67%), having a low incidence of early adverse events, not influenced by MELD (although average MELD scores ranged from 12 to 22, below the threshold of 24, usually associated with adverse post-transplant outcomes [15]).

An increase in blood transfusion during liver transplantation [16] and the need for veno-venous bypass [4] correlate with worse outcome. A recent study [17] reported that patients undergoing liver transplantation receive now significantly less blood transfusions, likely due to changes in surgical technique rather than changes in transfusion triggers over the study period. Likewise, avoiding excessive fluid administration and maintaining a relative hypovolemia has been recommended to minimize blood loss during hepatectomy in transplantation, and TEG was shown to allow a rapid on-site assessment of the functional clotting status, facilitating a selective use of blood components or drug treatments, leading to a net reduction in the need for blood products [18].

A single-center study of 500 consecutive liver transplants [19] reported a mean of 0.5 ± 1.3 units of PRBC per patient, with 79.6% of them not receiving any blood, which is in line with our patients’ needs (mean of 0.43 ± 0.96 units of PRBC per patient, with 81.1% nor receiving any transfusion). We believe that a specific surgical technique (i.e., caval preservation and TPCS) as well as a finely tuned anesthetic management (i.e., fluid restriction, blood salvage, liberal use of vasopressor drugs, and tailored coagulation management with thromboelastography) significantly reduced intraoperative blood loss and the need of blood products during liver transplantation in our study.

Coagulopathy in a cirrhotic patient is related to a reduction of coagulation factors, and the presence of anti-coagulants, thrombocytopenia, deficit of factor VII, and increased plasma levels of coagulation factor VIII and Von Willebrand factor [20], [21]. Conventional measurement of individual components involved in the coagulation process does not correlate with the risk of bleeding, and an attempt to correct the coagulopathy on the basis of deviations of each component has proven difficult and lead to an excessive volume administration. Alternatively, thromboelastometry has become a better tool, providing information on clot strength, and diagnosing and quantifying fibrinolysis, thus guiding the use of anti-fibrinolytic drugs and blood products. It may also diagnose platelet dysfunction and hypercoagulopathy, which in turn prevents unnecessary transfusions and inappropriate use of blood components [22], although a recent Cochrane systematic review [23] suggested that further trials are required to ascertain whether TEG can improve outcome. Some patients in the present study displayed coagulopathy and many had thrombocytopenia, which was severe in some cases, and yet they received few units of PRBC or platelets.

Liver transplantation with TPCS has been associated with reduction in the intraoperative use of blood products, preserved kidney function, shorter hospital stay, and lower 30-day postoperative mortality [24, 25]. Furthermore, TPCS can add benefits to the caval preservation in liver transplantation improving perioperative outcomes, particularly in higher-risk grafts and recipients [24], yet the debate over its usefulness or futility remains, and larger series will be needed to settle this subject [26, 27].

Due to inter- and intra-individual differences influencing the metabolism of tacrolimus and its narrow therapeutic index, monitoring of the drug trough levels is necessary and difficult to ascertain following liver transplantation [28]. Obtaining a target plasma level of tacrolimus before a steady state is achieved may be difficult to predict with a particular dose. The use of a Bayesian methodology to forecast the plasma levels of tacrolimus may help in predicting the results. In addition, the correlation between blood concentration and drug exposure can be improved by the use of several non-trough time points [29]. In the present study, proactive pharmacokinetic monitoring facilitated drug dosage and helped in obtaining stable plasma concentrations of tacrolimus after liver transplantation in an outpatient basis.

A review of the OPTN/UNOS database [30] identified 66,461 patients receiving a primary isolated cadaveric liver transplant in a 20-year period (1993–2012), and their median MELD was 17 at listing, and 20 at transplantation. The most recent published SRTR data reports a median MELD of 28 in the US [31]. In our series, MELD was 15.5 (MELD-Na 16.8) both at listing and at transplantation. These differences can be explained by the higher proportion of HCC among our recipient population and the much higher supply of cadaveric organs in Spain. An analysis of the Spanish National Transplant Organization (ONT) database for 2016 reflected that the listed patients who received a liver transplant—excluded deaths and drop outs—waited a median of 92 days. In the same period, patients in our series waited a median of 76 days, with similar waiting times in the previous four years.

Our ICU length of stay and the overall hospital length of stay are short for any liver transplantation program. An important factor that might have a positive effect in the prompt hospital discharge is the proximity of the patient’s home to the hospital. In our series, the patients living the farthest from the hospital were not more than 75 to 80 minutes away, which certainly helps lessen the burden on the patients and their families when considering leaving the hospital.

A single-center adult deceased donor liver transplantation program in the US showed a 90-day readmission rate of 46%, being their hospital length of stay a predictor of readmission, concluding that readmissions help identify patients more likely to have a worse outcome, but they may also provide an opportunity to intervene [32]. Yataco et al. [33] concluded that early discharge (fewer than 7 days after liver transplant) was not associated with a higher readmission rate, although a prolonged hospital stay after transplant was associated with an increased readmission risk. Our 30-day readmission rate was 34.9%, and it was significantly lower amongst the fast-track group, in line with these studies.

Transplant centers have different practices and philosophies that may influence outcomes in a manner difficult to identify and quantify. The elements of the fast-track pathway employed in the present study are commonplace and might be replicated and validated in liver transplant programs elsewhere. Although cost analysis is not part of the present paper, the decrease in hospital stay without an increase in morbidity and readmission has already proven to achieve significant cost savings. The present study demonstrates that fast-tracking from hospital admission to discharge can be implemented in a new liver transplantation program, can be accomplished without compromising patient’s safety, and can help reduce the use of health care resources.

Our study has several inherent weaknesses. The analysis of outcomes in this multistep protocol reflects real clinical conditions and is limited by the fact that we do not know which steps might have had a greater or lesser effect on the outcomes, which in turn limits our ability to systematically improve the protocol through practice-based evidence. Furthermore, by design, we don’t have a control group to determine if the outcomes reflect improvement or not, and we cannot provide historical data since our program is new; for that reason, we could only rely on previously published outcomes data to put ours in context. However, a recent prospective randomized study [34] showed that fast-track procedures reduced ICU stay after liver transplant from a median of 5 days in the control group to 2 days in the fast-track group and hospital stay from 28 to 18 days without adversely affecting prognosis.

Regarding the strengths, this is the first report of a large clinical series of patients being fast-tracked from liver transplant to discharge and showing the shortest reported length-of-stay (30 patients sent home on the 2nd post-liver transplant day), associated with our thorough enhanced recovery protocol after liver transplantation (which, as previously stated, has many steps previously validated in carefully conducted clinical studies). Overall, the success of any protocol is not necessarily determined by the details of each step, but by the systematic approach of consistently utilizing multiple steps that are based on previously published evidence. Our results may not necessarily be extrapolated to other centers with higher average MELD scores; however, we are confident that centers with similar conditions should be able to produce similar results.

## Abbreviations

DCD: Donor after Circulatory Death
ERAS: Enhanced recovery after surgery
MELD: Model for End-Stage Liver Disease
NASH: Non-alcoholic Steatohepatitis
PBC: Primary biliary cirrhosis
PTLOS: Post-Transplant Length of Stay
HRS: Hepato-Renal Syndrome
ICU: Intensive Care Unit
PRBC: Packed Red Blood Cells
TPCS: Temporary Portocaval Shunt
VCP: Vena Cava Preservation
HCC: Hepatocellular Carcinoma
HCV: Hepatitis C Virus
HBV: Hepatitis B Virus
